# Identification of Ischemic Regions in a Rat Model of Stroke

**DOI:** 10.1371/journal.pone.0004764

**Published:** 2009-03-10

**Authors:** Anke Popp, Nadine Jaenisch, Otto W. Witte, Christiane Frahm

**Affiliations:** Department of Neurology, Friedrich-Schiller-University, Jena, Germany; Julius-Maximilians-Universität Würzburg, Germany

## Abstract

**Background:**

Investigations following stroke first of all require information about the spatio-temporal dimension of the ischemic core as well as of perilesional and remote affected tissue. Here we systematically evaluated regions differently impaired by focal ischemia.

**Methodology/Principal Findings:**

Wistar rats underwent a transient 30 or 120 min suture-occlusion of the middle cerebral artery (MCAO) followed by various reperfusion times (2 h, 1 d, 7 d, 30 d) or a permanent MCAO (1 d survival). Brains were characterized by TTC, thionine, and immunohistochemistry using MAP2, HSP72, and HSP27. TTC staining reliably identifies the infarct core at 1 d of reperfusion after 30 min MCAO and at all investigated times following 120 min and permanent MCAO. Nissl histology denotes the infarct core from 2 h up to 30 d after transient as well as permanent MCAO. Absent and attenuated MAP2 staining clearly identifies the infarct core and perilesional affected regions at all investigated times, respectively. HSP72 denotes perilesional areas in a limited post-ischemic time (1 d). HSP27 detects perilesional and remote impaired tissue from post-ischemic day 1 on. Furthermore a simultaneous expression of HSP72 and HSP27 in perilesional neurons was revealed.

**Conclusions/Significance:**

TTC and Nissl staining can be applied to designate the infarct core. MAP2, HSP72, and HSP27 are excellent markers not only to identify perilesional and remote areas but also to discriminate affected neuronal and glial populations. Moreover markers vary in their confinement to different reperfusion times. The extent and consistency of infarcts increase with prolonged occlusion of the MCA. Therefore interindividual infarct dimension should be precisely assessed by the combined use of different markers as described in this study.

## Introduction

Focal cerebral ischemia results in typical pathophysiological events that evolve in time and space. These processes are not limited to the lesion itself but differentially affect perilesional and even remote areas (for review, see [1]). Experimental induction of a focal ischemia in rats by intraluminal suture-occlusion of the middle cerebral artery was first developed by Koizumi et al. [2] and has been further modified by Longa et al. [3] and by Nagasawa and Kogure [4]. Since then this simple and noninvasive model has been extensively used for studies of cerebral ischemic pathophysiology and therapeutic interventions that focus on the protection of periinfarct tissue.

Besides the duration of occlusion of the middle cerebral artery (MCA), that is known to mainly determine the degree of injury [4], [5], other experimental factors influence the outcome following this surgery [3], [5]–[9]. Furthermore occlusion of the MCA requires manipulation of the internal carotid artery (ICA) thereby territories supplied by branches of the ICA (hippocampus, thalamus, hypothalamus, and amygdala) might also be susceptible to ischemic damage [3], [10], [11]. Therefore a spatial and temporal identification of the infarct core as well as the discrimination of differently affected perilesional areas is a prerequisite for studies that want to correlate the extent of neuroprotection or the variability of post-ischemic gene regulation with a defined degree of injury.

Nowadays non-invasive techniques like MRI - that are routinely applied in humans - can be also used to define ischemic lesions in rodents over time [12], [13]. Nevertheless histological analyses are often applied simultaneously for a thorough infarct validation post-mortem with an additional advantage of a cellular resolution. Until now there have been no reports in which vital and histological stainings combined with several molecular markers have been assessed after MCAO over a wide range of reperfusion times to discriminate differentially affected ischemic regions. Here, we systematically evaluated a practical approach to identify areas that are differently affected by ischemia at various times (2 h, 1 d, 7 d, and 30 d) after a mild (30 min) or a severe (120 min) insult and at 1 d following a permanent MCAO. To visualize the infarct core 2,3,5-triphenyltetrazolium hydrochloride (TTC) staining (introduced by Liszczak et al. [14] and Bederson et al. [15]) was applied. Subsequently these samples were used for Nissl staining as well as immunohistochemistry. To evaluate spatiotemporal changes in perilesional but also in remote ipsi- or even contralateral areas [16] three molecular markers were used. Microtubule-associated protein 2 (MAP2) is a cytosceletal marker mainly localized to dendrites of neurons in the mature rat brain [17]. A diminished immunoreactivity of MAP2 has been found to be an early and sensitive marker of ischemic damage after permanent and transient focal stroke [18], [19]. The inducible heat shock proteins HSP72 and HSP27 are stress proteins known to be differently expressed after focal ischemia with regard to cell type, regional distribution, and injury-reperfusion times. HSP72 expression is mostly restricted to perilesional neurons, whereas HSP27 has been reported to be widely inducible mainly in glial cells of perilesional and remote areas [20]. Therefore these markers were selected out of a wide range of potential molecular markers of ischemic injury [21], [22].

Hereafter differently impaired regions were separated into (1) the infarct core [necrotic damage], (2) perilesional areas [non-necrotic areas affected by a decreased blood flow mainly in territories of the MCA and branches of the ICA] and (3) remote areas [non-ischemic but indirectly affected regions].

## Materials and Methods

### Focal stroke model – transient occlusion of middle cerebral artery (MCAO)

Male Wistar rats weighing 270–300 g were used. All animal procedures were approved by the local government (Thueringer Landesamt, Weimar, Germany) and conformed to international guidelines on the ethical use of animals.

Transient MCAO was induced according to Nagasawa and Kogure [4]. Rats were anesthetized with 2.5% isoflurane in a mixture of N_2_O/O_2_ (70%∶30%). A commercial 4-0 monofilament nylon suture coated with silicone rubber on the tip [(0.35±0.02 mm diameter; 35SPRe); Doccol Corp, USA] was introduced through an incision of the right common carotid artery into the ICA to occlude the origin of the right MCA for 30 min (n = 32), 120 min (n = 32) or permanently (n = 11). During surgery body temperature was maintained at a physiological level. Cerebral blood flow was measured by laser Doppler flowmetry (LDF) (Peri Flux System 5000, Perimed, Sweden) in cortical areas supplied by the MCA (2 mm posterior and 6 mm lateral to bregma) from before the onset of MCAO until 15 min after reperfusion. Sham operated animals (n = 12) underwent the same procedure but without occluding the MCA.

### Sample preparation

Brains were removed at various times after reperfusion (30 min MCAO: 2 h, 1 d, 7 d, and 30 d; 120 min MCAO: 2 h, 1 d and 7 d; permanent MCAO: 1 d). Using a rat brain slicer (Rodent Brain Matrix, Adult Rat, Coronal Sections, ASI Instruments, USA) 3 mm coronal sections were dissected, cutting at distances of 2, 5, 8, and 11 mm from frontal pole. Section between 5 and 8 mm (bregma 1.0 to −2.0 mm±0.5 mm according to Paxinos and Watson [23]), including the main portion of the infarct, was snap frozen and stored for further analyses. Adjacent sections between 2 and 5 mm (bregma 4.0 to 1.0 mm±0.5 mm; anterior) and 8 and 11 mm (bregma −2.0 to −5.0 mm±0.5 mm; posterior) were used for characterization of the infarct.

### TTC staining

After sectioning, slices were immediately immersed in 2% TTC in 0.9% NaCl at 37°C for 10 min for vital staining, photographed, and transferred in 4% PFA for immersion fixation for 24 h. After dehydration in 30% sucrose sections were cut into coronal slices of 30 µm on a microtome (Microm GmbH, Germany) for further use.

### Nissl staining

Slices from the anterior and posterior sections (previously stained in TTC) where mounted onto slides and stained with 0.2% thionine.

### Immunohistochemistry (MAP2, HSP72, HSP27)

Free floating sections were treated with 0.24% H_2_O_2_ before the antibodies (mouse anti-MAP2 1∶1000, M1406, Sigma, Germany; mouse anti-HSP70 1∶500, SPA-810, Stressgen, USA; goat anti-HSP27 1∶1000, sc-1049, Santa Cruz Biotechnology, USA) were applied in TBS containing 3% normal donkey serum and 0.2% Triton X-100. Sections were incubated with the antibodies at 4°C overnight and further processed by the Vectastain Elite ABC Kit (Vector Laboratories, USA) using either a donkey anti-mouse or a donkey anti-goat biotinylated secondary antibody (Dianova, Germany). Finally, immunoreactivity was developed in 3,3′-diaminobenzidine tetrahydrochloride (DAB, Sigma, Germany). For co-labeling of HSP72 and HSP27 the primary antibodies were simultaneously applied and detected by fluorescence-labeled secondary antibodies (Alexa488-donkey anti-goat, Rhodamin-donkey anti-mouse; Molecular Probes, Inc., Invitrogen, USA). Slices were analysed via confocal microscopy (LSM 510, Carl Zeiss MicroImaging GmbH, Germany).

## Results

### General results

All animals with a dropdown of LDF signal to ≤40% were included in this study. The LDF signals were 27,7%±8,9% of control values (before insertion of the filament) following occlusion of the MCA and recovered to 80,7%±13,1 during reperfusion. There was a very low mortality rate following 30 min MCAO [9% overall including all reperfusion times (3 out of 32 rats)]. Following 120 min MCAO the mortality rates increased over time [2 h: 12% (1 out of 8); 1 d: 20% (2 out of 10); 7 d: 43% (6 out of 14)]. Because of the still higher mortality rate in the 120 min MCAO group within 30 days this reperfusion time was omitted. Until 1 d following permanent MCAO 27% (3 out of 11) of operated rats died.

All MCAO rats displayed an infarct whereby the infarct size varied. Following mild MCAO (30 min) the infarct always included the striatum and frequently the neocortex. After a severe MCAO (120 min) the necrotic area extended over the striatum to large portions of the adjacent neocortex, amygdala, and hypothalamus. Permanent MCAO produced the largest and most consistent infarcts with an infarct core always including striatum, neocortex, amygdala, and hypothalamus. The formation of edema was strongest under these conditions.

### TTC staining

**Figure 1:**
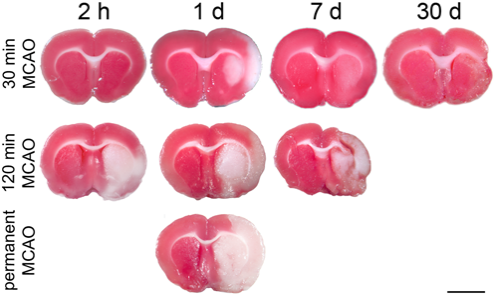
TTC-stained brain sections at various reperfusion times following transient (30 min and 120 min) and permanent MCAO. TTC method reliably delineates the infarct core at 1 d after 30 min MCAO. After 120 min and permanent MCAO the infarct core was clearly detectable at all investigated times. Scale bar: 5 mm.

Following 30 min MCAO TTC staining did reliably delineate the infarct (pale areas versus non-injured deep red colored tissue) at 1 d after reperfusion (Fig. 1). No clear demarcation of injured areas was found at all other investigated times. Following 120 min MCAO irreversible injured tissue could be clearly distinguished from 2 h (4 h after the onset of ischemia) up to 7 d after reperfusion (Fig. 1). Demarcation of the infarct 1 d after permanent MCAO was clearly visible (Fig. 1).

### Nissl staining

**Figure 2:**
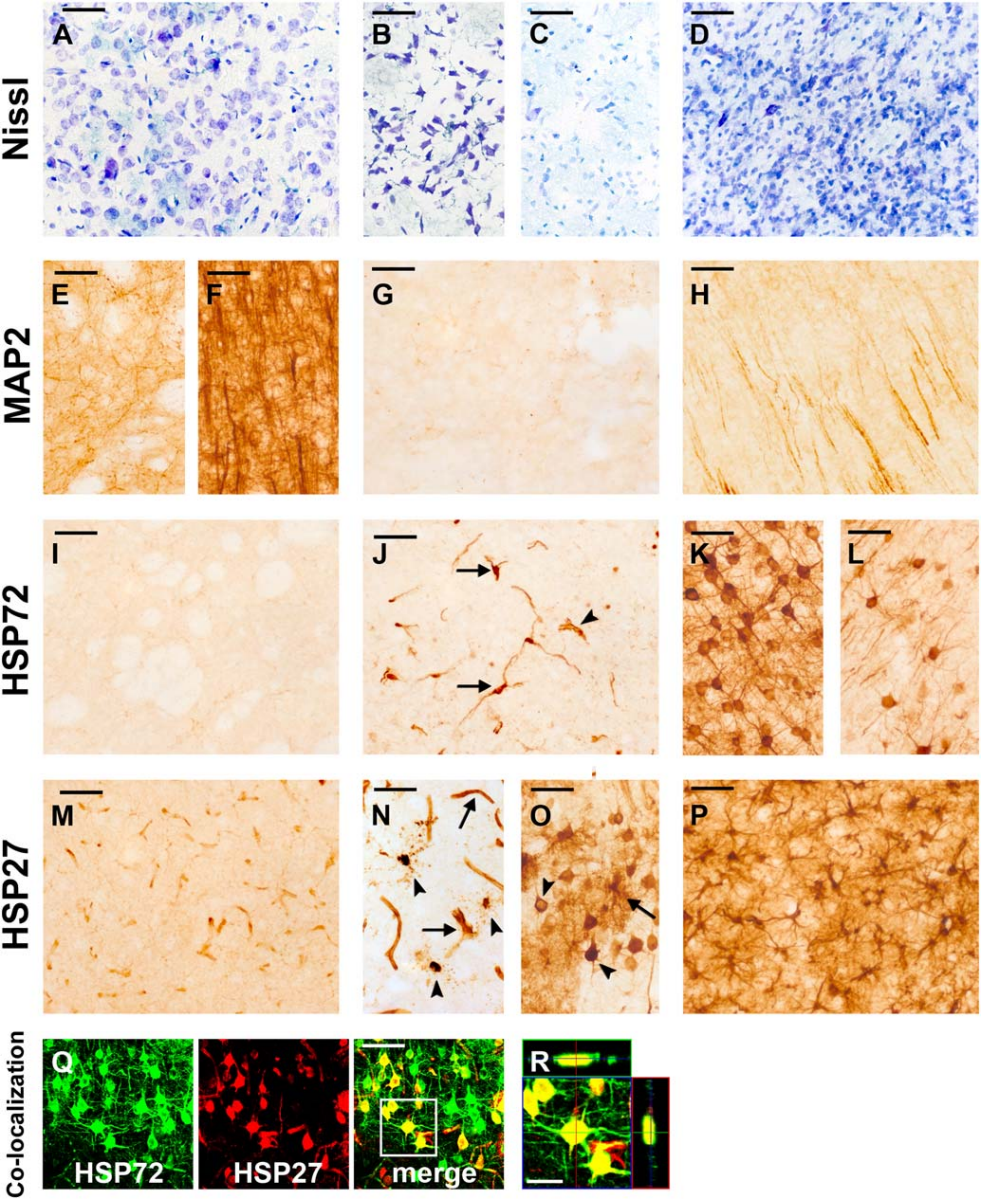
Thionine staining (A–D) of the contralateral (A) and the ipsilateral striatum after 30 min MCAO (B–D). Cells displaying aberrant morphology after 2 h reperfusion (B), major cell loss was found at 1 d (C) and a massive infiltration of microglia and astrocytes occurs at 7 d after reperfusion (D). MAP2 immunoreactivity (E–H) in the contralateral striatum (E) and neocortex (F). Absent MAP2 immunoreactivity in the infarct core at 1 d after 30 min MCAO (G). Attenuated MAP2 immunoreactivity in the ipsilateral neocortex at 1 d after 30 min MCAO (H). HSP72 immunoreactivity (I–L) in the contralateral (I), the ipsilateral striatum (infarct core) at 1 d after 30 min MCAO with HSP72-positive bipolar (arrows) and endothelial cells (arrowhead) (J) and HSP72-positive neurons in the ipsilateral neocortex (perilesional zone) at 1 d after 30 min (K) and 120 min MCAO (L). HSP27 immunoreactivity after 30 min MCAO (M–P) in the contralateral striatum with HSP27 expression in endothelial cells (M), the ipsilateral striatum (infarct core) at 1 d after reperfusion with an upregulated HSP27 expression in endothelial cells (arrows) and microglia (arrowhead) (N), HSP27-positive astrocytes (arrow) and neurons (arrowheads) in the ipsilateral neocortex 1 d after reperfusion (O). HSP27-positive astrocytes at 7 d after reperfusion in the ipsilateral neocortex (perilesional zone, P). Co-localization of HSP72 and HSP27 at 1 d after 30 min MCAO (Q, R), revealing co-expression of both proteins in most neurons. The framed area is enlarged in R. Scale bar A–Q: 50 µm, R: 20 µm.

**Figure 3:**
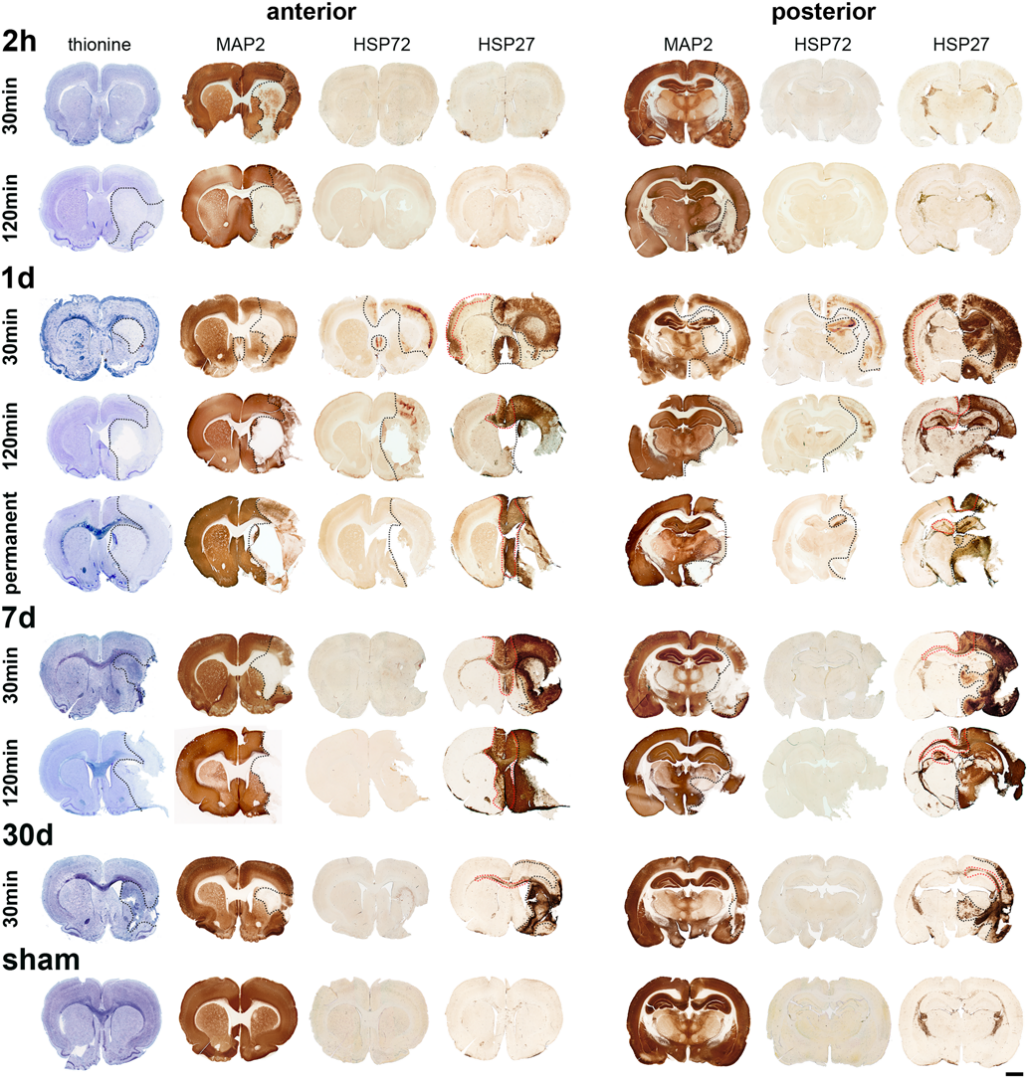
Histological (thionine) and immunohistochemical (MAP2, HSP72, HSP27) characterizations of infarcts induced by a transient (30 and 120 min) and permanent MCAO. Occlusion of the MCA for 30 min always leads to an irreversible injury (infarct core) in the striatum and frequently in parts of the neocortex. The neocortex is also part of the perilesional region which also might include hippocampus, amygdala, thalamus, and hypothalamus. An irregular impairment of remote areas also occurs (cingulate and retrosplenial cortex, septum, and all contralateral affected regions). Following 120 min MCAO striatum, neocortex, amygdala, and hypothalamus are part of the infarct core. Impaired perilesional zones include hippocampus, thalamus, and hypothalamus. The ipsi- and contralateral cingulate and retrosplenial cortex and the contralateral hippocampus as well as the septal area were identified as remote affected regions. Permanent MCAO leads to infarct cores always comprising the striatum, neocortex, amygdala, and hypothalamus. Further impaired regions are similar as identified following 120 min MCAO. Rare cerebrovascular anatomies like a unilateral origin of the ACA could also lead to ischemic changes in usually remote areas like cingulate and retrosplenial cortex (1 d following 30 min MCAO). Black dotted lines indicate the infarct core and perilesional affected regions, whereas red dotted lines display remote effects. Please note that a cellular resolution can not be provided by this overview, for this purpose please refer to Fig. 2. Scale bar: 2 mm.

Intact cells (Fig. 2A) could be found in sham operated animals as well as all non-affected regions following ischemia. After mild as well as severe ischemia cells with aberrant morphology were visible as early as 2 h of reperfusion (Fig. 2B). These cells were triangular in shape mostly exhibiting a dark staining due to condensation of cytoplasm and karyoplasm (see also Oechmichen and Meissner [24]). A clear demarcation of infarcted areas after 30 min MCAO was not obvious until 1 d of reperfusion where affected tissue was brightened (Fig. 2C, 3). From day 7 of reperfusion on a glial scar surrounded (or sometimes even masked) the infarct revealed by a pronounced Nissl stain (Fig. 2D, 3). After occlusion of the MCA for 120 min thionine revealed brightened thus injured areas as early as 2 h after reperfusion. Following 1 d of permanent MCAO the infarct was also clearly demarcated with this staining.

### Immunohistochemistry (MAP2, HSP72, and HSP27)

The extent of neuronal injury could be identified by an absence as well as an attenuation of MAP2 immunoreactivity. After 30 min MCAO a diminished MAP2 expression could be detected as early as 2 h after reperfusion in striatum and neocortex. From 1 d of reperfusion on lack of MAP2 (necrotic areas) could be observed mainly in the striatum and occasionally in parts of the neocortex (Fig. 2G, 3). Following a mild MCAO typical perilesional zones like neocortex, hippocampus, amygdala, thalamus, or hypothalamus displayed an attenuated staining (Fig. 2H, 3). Occlusions of the MCA for 120 min as well as a permanent closure of the MCA led to an infarct core (characterized by absent MAP2) including striatum, neocortex, amygdala, and hypothalamus. Perilesional sites with attenuated MAP2 staining were rarely detectable (Fig. 3).

In agreement with many other studies [20], [25]–[28] we detected HSP72 in the ischemic core exclusively in capillaries and in cells having bipolar processes (Fig. 2J). HSP72 expressing neurons were restricted to 1 d after reperfusion, regardless of the occlusion time (Fig. 3). A mild MCAO led to HSP72 positive neurons mainly located in the ipsilateral cortical layer IV (Fig. 2K) but to a lesser extent also in adjacent layers. Moreover HSP72 expression could also be found in neurons of striatum, hippocampus, thalamus, and hypothalamus (Fig. 3). Two rats out of 49 (one is displayed in Fig. 3) also exhibited HSP72 expression accompanied by attenuated MAP2 staining in the ipsi- and even the contralateral cingulate and retrosplenial cortex and the septum (Fig. 3). HSP72 expression following severe ischemia was markedly attenuated and spaciously restricted (Fig. 2L). Positive neurons were mainly located at the periphery of the core zone (Fig. 3). After permanent MCAO HSP72 expression was hardly detectable in the neocortex and restricted to very few neurons adjacent to the infarct core (Fig. 3). Some rats displayed a hippocampal HSP72 expression in the pyramidal cell layer.

HSP27 was detectable at 1 d of permanent MCAO and from 1 d of reperfusion on after mild as well as severe ischemia (Fig. 3). Regions in which HSP27 expression was induced were similar when comparing different occlusion times of the MCA (Fig. 3). In line with other studies the infarct core displayed HSP27 stained endothelial cells and very few microglia [20], [29] (Fig. 2N). Infrequently, HSP27 positive astrocytes were also detected there (double staining with GFAP, data not shown). Reactive astrocytes forming the glial scar strongly and robustly expressed HSP27 (Fig. 3). In addition HSP27 was found to be spaciously distributed in the striatum and the neocortex (Fig. 2P) as well as in perilesional hippocampal, thalamic, and hypothalamic regions. HSP27 was further induced in remote regions like the ipsi- and contralateral cingulate and retrosplenial cortex, the septum, the corpus callosum, the contralateral neocortex, and hippocampus, implying a widespread activation of astrocytes there (Fig. 3). Though HSP27 was mainly expressed in astrocytes it was also found in neurons at 1 d after infarction (Fig. 2O; see also Kato et al. [20] and Currie et al. [30]). Neuronal expression was by far higher after a mild ischemia, predominantly in the neocortex but also obvious in the hippocampus, the thalamus, and the hypothalamus (data not shown). Double immunofluorescence stainings were performed to answer the question if HSP27 positive neurons also express HSP72 and indeed a co-localization could be revealed in most neurons (Fig. 2Q, R).

Slices from sham operated animals were adequately stained. We and others found HSP27 constitutively expressed in few astrocytes (mainly in fibre tracts) [31] and in vessels [32]. All other tested markers were negative (HSP72) or showed a normal staining pattern (MAP2; Fig. 3). The same could be observed in all non-affected regions following ischemia (Fig. 2E, F, I, M).

## Discussion

The TTC method rests on the functioning of mitochondrial enzymes [14]. After a mild ischemia (30 min MCAO) conventional TTC staining was applicable at 1 d of reperfusion exclusively. After 120 min and permanent MCAO the infarct core was clearly detectable at all investigated times. The earliest time of a reliable TTC stain, as reported in the literature, is around 3 h after onset of the MCAO [14], [33]. The latest is, as shown here, depending on the duration of the occlusion. A clear advantage of this method is its easy applicability, the immediate availability of results and its suitability for infarct volume evaluation [15], [33]–[36]. Nissl stain exhibited cells with aberrant morphology as early as 2 h after reperfusion onset whereas an identification of the ischemic core was reliable from 1 d of reperfusion on. Nissl methods are easy applicable and widely accepted for evaluation of the infarct volume [36]. Unfortunately they do not cover very early reperfusion times after a mild ischemia and are not suitable for identification of perilesional affected tissue.

**Figure 4:**
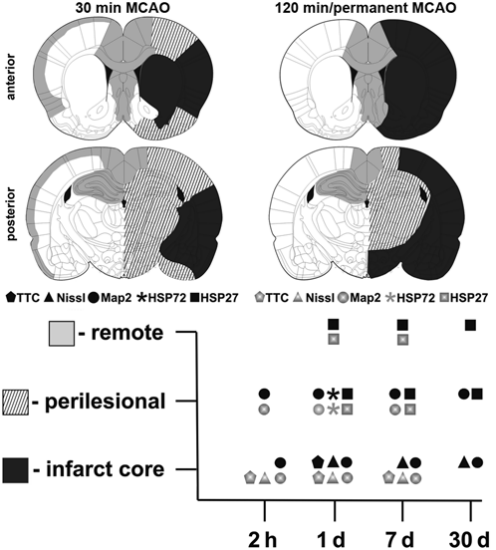
Overview of differently affected regions following 30 min, 120 min, and permanent MCAO (black: infarct core; striped: perilesional regions; light grey: remote areas). The maximum expansion of each region is displayed, whereby 120 min and permanent MCAO revealed the same maxima. Additionally staining procedures used in this study are displayed relating to their applicability to detect these differently affected regions at various reperfusion times (2 h, 1 d, 7 d, 30 d).

By using immunohistochemical markers not only the infarct core but also perilesional and remote affected regions could be discriminated (Fig. 4). Besides areas supplied by the MCA, like striatum and neocortex, other areas which not seem to be primarily connected to the MCA were also found to be injured. The amygdala was often affected due to blood supply by branches of the MCA as well as ICA [37]. Hippocampal damage has already been shown earlier [38], [39] possibly due to involvement of the posterior cerebral artery (PCA) which is connected to the ICA via the posterior communicating artery (PCOM) [2]. Blood supply of the thalamus is inter alia mediated by branches from the initial part of the MCA [40], wherefore thalamic damage after MCAO was not uncommon. The hypothalamus, also often affected, is supplied by the hypothalamic (HTA) and anterior choroidal arteries (AChA), both arising from the ICA proximal to the MCA and therefore might be also blocked by the filament [11]. Two animals out of 49 (one is displayed in Fig. 3) showed neuronal damage in the ipsi- and contralateral cingulate and retrosplenial cortex and septum (attenuated MAP2 and HSP72 induction), regions which are supplied by the ACA. Normally these areas are not ischemic because of collateral blood flow from the left ACA and the pterygopalatine artery (PPA) [3], and are therefore classed with remote regions. But very few rats seem to have a unilateral origin of the ACA as also sometimes seen in humans.

MAP2 not only identifies the infarct core but also reveals ischemic damage in perilesional zones (defined by attenuated MAP2 staining) very sensitively. After mild MCAO in general a relatively broad area of partial MAP2 degradation was revealed. Following severe as well as permanent ischemia the infarct core was extended whereas perilesional regions were narrow. Since MAP2 is involved in maintaining the structural integrity of the neuronal cytoskeleton [41] the observed post-ischemic loss of MAP2 immunostaining reflects cytoskeletal degradation. Subtle changes in dendritic integrity might imply functional modifications of synaptic circuits in respective regions. Though we found MAP2 is an excellent marker at all investigated post-ischemic times its early sensitivity is of obvious advantage and has been confirmed by several studies [18], [42].

Transient post-ischemic HSP72 expression might start as early as 3 h after brain injury persisting for about 7 d [20], [28], [43], [44]. Here we found HSP72 to be expressed at 1 d after the onset of permanent MCAO as well as at 1 d after reperfusion. In regions adjacent to the infarct we revealed an exclusive neuronal HSP72 expression in line with Li et al. [27], [28] and Kinouchi et al. [25] but in contrast to others who detected HSP72 also in astrocytes and microglia [20], [43], [45], [46]. These conflicting results might be due to experimental deviations or specific tissue handling. After mild ischemia this neuronal induction of HSP72 was strong and broad in perilesional areas whereas after severe and permanent ischemia faintly stained neurons could only be detected in a narrow perilesional zone close to the necrotic area. It is well accepted that HSP72 expressing neurons have a good prognosis to survive [26], [28], [47], [48] and therefore these regions are often considered as ischemic penumbra [20], [21], [25]–[27], [47].

Our results confirmed a post-ischemic upregulation of HSP27 after focal ischemia mainly in reactive astrocytes [20], [29] both after transient and permanent cerebral ischemia. In line with other studies HSP27 expression was found to be induced from 1 d of reperfusion on [20], [29], but may start as early as 12 h after ischemia [49]. HSP27 expression in reactive astrocytes participating on astrogliosis seems to be very robust (up to 30 d) whereas its expression in activated astrocytes might be more transient. Ipsilateral HSP27 positive astrocytes in regions without neuronal damage might be activated via spreading depression (SD) outside the territories supplied by the MCA or branches of ICA [21], [30], [50]. A contralateral HSP27 expression was also found by us and others [20], [30]. Though most studies did not find SD to be involved in this phenomenon [51], [52] there is evidence of a contralateral impairment [53]. One of the two animals (out of 49 processed for MCAO) exhibiting neuronal damage in the contralateral cingulate and retrosplenial cortex additionally displayed a neocortical HSP27 expression that might be induced by SD. This possibility is strengthened by the fact that the contralateral ischemic cortex might be the origin of SD in this animal (Fig. 3). Frequently found HSP27 induction in contralateral areas close to the midline (Fig. 3) indicates edema pressure as one possible reason.

For the bilateral hippocampal induction of HSP27 one might discuss several causes. Though a bilateral transient impairment of cerebral blood flow has to be considered we think that secondary responses to ischemia are more relevant (e.g. post-ischemic depolarization, diachisis, transcallosal effects, overexcitations, transsynaptic effects, bilateral blood brain barrier opening, edema) [21], [47], [54]–[56]. The general stress response following these processes is moreover in agreement with the well documented expression of immediate early genes [55]. Given the importance of astrocyte functioning to the microenvironment of the CNS and the close structural and physiological association to neurons, it is apparent that alterations in the astrocytic physiology could have profound implications [21], [57]–[59].

Additionally to the HSP27 expressing astrocytes HSP27 positive neurons were detected. Double immunofluorescence stainings of HSP27 and HSP72 revealed a co-staining in the majority of labeled neurons. The induction of two heat shock proteins strengthens the possibility of these perilesional affected neurons to survive. All in all HSP27 is a useful marker to sensitively detect perilesional and far remote affected regions over a large period of time.

Though a standardized protocol for infarct induction was used individual deviations occur inter alia due to varying cerebrovascular anatomy. The variability of infarcts as well as the involvement of perilesional and remote affected regions was most pronounced after mild MCAO (Fig. 4). With prolonged occlusion of the MCA the infarct extent increased and the infarcts were more consistent. That demonstrates the need of a thorough post-ischemic characterization of individual animals, especially when neuroprotective or gene expression studies (e.g. cDNA arrays, qPCR, western blotting) are planned. As shown in this study this could be achieved by using different markers varying concerning their restriction to certain reperfusion times and their sensitivity in detecting differently affected regions and cell types.
